# Exploring the Support Needs of Family Caregivers of Patients with Brain Cancer Using the CSNAT: A Comparative Study with Other Cancer Groups

**DOI:** 10.1371/journal.pone.0145106

**Published:** 2015-12-17

**Authors:** Samar M. Aoun, Kathleen Deas, Denise Howting, Gabriel Lee

**Affiliations:** 1 School of Nursing, Midwifery & Paramedicine, Curtin University, Western Australia, Australia; 2 St John of God Hospital, Subiaco, Western Australia, Australia; 3 School of Surgery, University of Western Australia, Western Australia, Australia; Shenzhen Institutes of Advanced Technology, CHINA

## Abstract

A substantial burden is placed on family caregivers of patients diagnosed with brain cancers. Despite this, the support needs of the caregivers are often under-recognised and not addressed adequately in current routine and patient centred clinical care. The Care Support Needs Assessment Tool (CSNAT) is a validated instrument designed to systematically identify and address caregiver needs. It has been trialled in an Australian palliative care community setting using a stepped wedge cluster design involving 322 family carers of terminally ill patients. The current article reports on a subset from this trial, 29 caregivers of patients with primary brain cancer, and compares their profile and outcomes to those of other cancer groups. Caregiver strain was assessed using the Family Appraisal of Caregiving Questionnaire, caregiver physical and mental wellbeing using SF12 and caregiver workload using a questionnaire on support with activities of daily living (ADL). In comparison to caregivers of patients with all other cancers, the primary brain cancer group had significantly higher levels of caregiver strain, lower levels of mental wellbeing and a higher level of ADL workload. Their physical wellness also deteriorated significantly over time. An action plan approach led to practical solutions for addressing highlighted concerns. Four themes evolved from the family caregivers’ feedback interviews: The extremely challenging caregiver experience with brain cancer; the systematic and practical approach of the CSNAT during rapid changes; connection with health professionals, feeling acknowledged and empowered; and timely advice and assurance of support during the caregiving journey. This preliminary study has demonstrated that the CSNAT provides a practical and useful tool for assessing the support needs of family caregivers of patients with brain cancer and has provided the basis for a larger scale, longitudinal study that allows a more detailed characterisation of the evolving caregiver needs at different stages of the disease.

## Introduction

The impact on family caregivers when providing home based family caregiving for the terminally ill is well documented to have substantial physical, social and psychological consequences [1–4], with extensive literature reporting these negative effects for caregivers of people with life limiting illnesses including brain tumours [5–8]. Provision of good support during caregiving, has been shown to improve family caregivers’ psychological outcomes [1,9–11] whilst identifying and addressing concerns early, leads to better carer health outcomes [1,10]. Nevertheless, adequate assessment of family caregivers’ support needs by health professionals is often hindered whilst focussing primarily on the care recipient, resulting in informal and undocumented needs assessment [5,12–15]. Moreover there is often reluctance by family caregivers to express their own needs [1,5,13].

In particular, caregivers of patients with brain cancer deserve further study. Despite the advent of newer chemotherapy agents, the median survival of patients diagnosed with glioblastomas remains approximately 12 months [16]. Similarly, patients who develop brain metastases have a comparable median survival, dependent on the primary cancer of origin [17]. Thus the diagnosis has catastrophic consequences and implications for both the patients and their families. Due to the sudden ‘crisis’ onset, an often rapid progression of the disease and the uncertainty of recovery [5,7,8,13,15], family caregivers of people with brain cancer often describe their caring experiences as physically and mentally challenging [8,15,18–20]. Unlike other cancers, the brain cancer trajectory encompasses significant cognitive impairment and neuro-behavioural changes as well as the physical symptoms associated with cancer, requiring a ‘unique’ level of caregiving [5,7,20]. People with brain cancer, in particular those with glioblastomas, can rapidly progress to distinctive neuro-oncological symptoms and physical deterioration [5,8,15,21] including personality changes requiring need for support, a high level of assistance with personal daily living tasks, problem solving and decision making or advocacy [8,15,20,22].

Recent studies have reported people with brain cancer and their family caregivers also suffer from social stigmatization associated with neuro-cognitive deficits [8,15]. Management of physical symptoms, cognitive impairment and neuro-behavioural changes in individuals with brain cancer is crucial, however addressing their family caregivers’ psychosocial issues is essential to prevent negative effects on their own physical and mental health outcomes [15]. As people with brain cancer are mainly cared for by their family at home during their illness, their neuro-cognitive symptoms and associated challenging behaviours closely affect their family members’ distress level and quality of life [5,8,21]. Targeted support with more effective interventions to accurately assess the unique support and palliative care needs of families living and caring for someone with brain cancer is essential to ease the stress and burden not met by current models of care [6,8,21].

Suitable tools are needed for assessment of family caregivers’ support needs in end-of-life home care [23,24] and in particular during the uncertainty between diagnosis, often rapid progression and end-of-life care in brain cancer [6–8,13,15,19].

### The Carer Support Needs Assessment Tool (CSNAT)

The Carer Support Needs Assessment Tool (CSNAT) is a validated evidence based tool used to identify family carer support needs in a systematic way, rather than the existing ad-hoc manner. As such the tool also serves as a supportive carer intervention and is carer-led, but facilitated by the health professional [14,23]. The CSNAT adopts a screening format, structured around 14 broad support domains. This format allows it to be brief but also comprehensive, enabling caregivers to identify the domains in which they require further support which can then be discussed with health professionals. Each item represents a core family carer support domain in end of life home care, and these domains fall into two distinct groupings: those that enable the family caregiver to care and those that enable more direct support for themselves. There are four response options for each of the 14 CSNAT items that allow family caregivers to indicate the extent of their support requirements for each domain: no more, a little more, quite a bit more, or very much more (14).

The CSNAT was trialled using a stepped wedge cluster trial in Silver Chain (a large community based service provider in Western Australia) with 322 family caregivers of terminally ill people (cancer and non-cancer) and 44 nurses. The intervention group showed significant reduction in caregiver strain relative to controls (p = 0.018, d = 0.35) [25] and feedback of family caregivers [26] and nurses [12] using the CSNAT was positive. Since the CSNAT appeared to offer a practical approach to assessing and addressing family caregiver needs in the general cancer field, it was deemed important to assess the extent to which the tool would be appropriate for use in specific cancers and test the suitability of the CSNAT with family caregivers of people living with brain cancer in the community, across the caring experience and not only at end of life.

### Objectives

To compare the profile and differences in wellbeing outcomes of family caregivers of people with brain cancer with those of people with all other cancers who participated in the CSNAT intervention and to assess the feasibility of the CSNAT as an intervention to identify and address support needs of family caregivers of people with brain cancer in home-based palliative care.

## Methods

The study was approved by the Curtin University Human Research Ethics Committee (HR 24/2011) and the Silver Chain Human Research Ethics Committee (EC App 068). All participants provided written informed consent to participate in this study and the two ethics committees approved this consent procedure.

A stepped wedge cluster trial was conducted in Perth, Western Australia, in three sites of the Silver Chain Hospice Care Service (SCHCS) in 2012–14, involving family caregivers of terminally ill people with cancer and non-cancer diagnoses. The detailed methodology, the description of the service and the outcome measures, and the interview questions have been described in previous publications [25,26]. This study focuses on a subset of the larger trial, specifically family caregivers of people with primary brain cancer, and makes comparisons with two other groups, brain metastases and all other cancers. There was a total of 29 primary brain cancer cases (9 in the control group and 20 in the intervention group) at baseline, and 18 at follow up (5 in the control group and 13 in the intervention group). Attrition rate between the two time periods was 38%, mainly due to patient deaths.

### Data Collection

Outcome measures were collected pre- and post-intervention by the researcher by telephone. Family caregivers’ support priorities were obtained through the set of items on the CSNAT, during nurses’ visits. Feedback from family caregivers was obtained via semi-structured telephone interviews at the end of the intervention period. Family caregivers were considered to have concluded the study if they have completed two CSNAT contacts with the nurse (2–3 weeks apart).

### Description of the intervention

The intervention consisted of the following steps:

- The CSNAT tool is introduced to the family caregiver by the nurse
- The family caregiver is given time to consider which domains they require more support with
- An assessment conversation takes place where the nurse and family caregiver discuss the domains where more support is needed to clarify the specific needs of the family carer including which are their priorities
- A shared action plan is made where the family caregiver is involved in identifying the type of input they would find helpful (rather than delivery of ‘standardised’ supportive input that the service is able to deliver)
- A shared review is planned within 2–3 weeks

### Outcome Measures

The primary outcome was caregiver strain and distress as measured by the 2 subscales of the Family Appraisal of Caregiving Questionnaire (FACQ-PC) where strain has 8 items and distress has 4 items [27]. Psychometric analyses demonstrate good construct validity. Internal reliability estimates range from 0.75–0.86. Scores range from 5 = strongly agree to 1 = strongly disagree.

Secondary outcomes were caregiver mental and physical wellbeing as measured by SF-12v2 [28] and caregiver workload as measured by caregiver assistance with Activities of Daily Living [25]. The SF-12v2 consists of 12 questions; relating to: physical health problems, bodily pain, general health perceptions, vitality (energy/fatigue), social functioning, role limitations and general mental health (psychological distress and psychological well-being). Reliability estimates range from 0.93 to 0.95 [28]. Caregiver workload was measured by the nature and extent of assistance provided by the family caregiver with a range of Activities of Daily Living (ADLs, such as feeding and toileting). Scores are: 4 = assistance all of the time; 3 = assistance most of the time; 2 = occasional assistance; 1 = no assistance required.

### Analysis

All statistical analyses were conducted using SPSS 22. Statistical significance was determined at an alpha value of 0.05. Analyses of this trial were on a per protocol basis. Continuous variables are reported as mean ± standard deviation and categorical variables are reported as n (%). Baseline differences between groups were assessed using Mann-Whitney U Tests for continuous variables and Chi-square tests or Fisher’s Exact Test (where cell sizes were less 5) for categorical variables. Due to the small numbers in the brain cancer control group, comparisons between intervention and control groups were not possible. However, a comparative analysis was undertaken between the brain cancer group and all other cancers for the outcome variables, both in the intervention arm of the study. Also a comparison was undertaken for three groups, including a group with brain metastases, for the demographic variables.

Data from the interviews with caregivers were subjected to a thematic analysis [29,30] and was supported by the NVivo 10 software programme. The interviews were audio-recorded, and thorough note-taking of interviews were verbatim. Transcribed interviews were read and re-read to identify key words and phrases that were then grouped into categories labelled with codes. To enhance the credibility of findings, the interviewer was involved in the analysis process so that consideration of the nonverbal context was assured. Themes emerging after comparisons within and among individual interviews identified key messages. These themes were initially identified independently, with differences resolved by discussion and by returning to the data. Exemplars are provided to explain themes and how interpretations have been reached [30].

## Results

Findings presented in this section relate to: a description of the profile of all study participants in three groups (primary brain cancer, brain metastases and all other cancers); a comparison of outcome variables between brain cancer and all other cancers (including metastases) for the intervention group; the identified support needs of family caregivers and actions taken by the service to provide support; and the family caregivers’ experiences in using the CSNAT, summarized in four themes.

### Participants’ characteristics

Table 1 presents a profile comparison between three groups who completed the study and includes those in both the control and intervention arms of the study: Primary brain cancer (n = 29), brain metastases (n = 30) and all other cancers not including the first two groups (n = 441). Primary brain cancer was the sixth most predominant cancer consisting of 6% of all cancers in this study, with the first five most predominant cancers being: Lung (22.4%), breast (9.6%), colorectal (8.8%), prostate (8%) and pancreas (7.2%). The primary cancer of people with brain metastases consisted mainly of: Lung (48.4%); breast (29.0%); melanoma (9.7%); and colorectal (6.5%).

**Table 1:** Profile of family caregivers and people with brain cancer compared to brain metastases and all other cancers at baseline.

Characteristics	Primary brain cancer		Brain metastases		All other cancers	
N	29		30		441	
	n	(%)	n	(%)	n	(%)
**Family caregiver**						
**Age**						
Mean (SD)	56.7	(11.21)	61.4	(11.78)	62.4	(12.64)
Median (min., max.)	60.0	(29, 73)	61.5	(35,82)	64.0	(20, 92)
**Gender**						
Male	7	(24.1)	9	(30.0)	119	(27.0)
Female	22	(75.9)	21	(70.0)	322	(73.0)
**Marital status**						
Never married	1	(3.4)	2	(6.7)	27	(6.1)
Widowed	0		0		12	(2.7)
Divorced/separated	2	(6.9)	1	(3.3)	26	(5.9)
Married/de facto	26	(89.7)	27	(90.0)	376	(85.3)
**Cultural background**						
Australian	19	(65.5)	18	(60.0)	269	(61.0)
Other English speaking	8	(27.6)	8	(26.7)	122	(27.7)
Non English speaking	2	(6.9)	4	(13.3)	50	(11.3)
**Education**						
Primary	0		0		12	(2.7)
Secondary	15	(51.7)	14	(46.7)	248	(56.2)
Trade/diploma	8	(27.6)	5	(16.7)	99	(22.4)
Tertiary	6	(20.7)	11	(36.7)	82	(18.6)
**Employment**						
Paid employment	12	(42.9)	5	(17.9)	136	(33.8)
Retired/volunteer	12	(42.9)	20	(71.4)	213	(53.0)
Other (inc. home duties, carer, unemployed)	4	(14.3)	3	(10.7)	53	(13.2)
**Relationship** ^A^						
Spouse/partner	25	(86.2)	19	(63.3)	301	(68.3)
Son/daughter	2	(6.9)	9	(30.0)	94	(21.3)
Parent	0		0		7	(1.6)
Brother/sister	1	(3.4)	1	(3.3)	11	(2.5)
Other (e.g. niece, carer)	1	(3.4)	1	(3.3)	28	(6.3)
**People with cancer**						
**Age** ^B^						
Mean (SD)	60.6	(10.75)	70.7	(8.03)	70.8	(12.70)
Median (min., max.)	61.0	(36, 83)	71.5	(50, 85)	71.0	(28, 94)
**Gender**						
Male	21	(72.4)	16	(53.3)	258	(58.5)
Female	8	(27.6)	14	(46.7)	183	(41.5)
**Length of diagnosis (months)**						
Mean (SD)	17.6	(27.19)	15.7	(15.56)	25.9	(40.46)
Median (min., max.)	12	(1, 150)	10	(1, 60)	12	(.3, 420)
**Length of palliative care(months)**	^C^					
Mean (SD)	3.7	(3.34)	2.6	(3.81)	3.5	(5.33)
Median (minimum, maximum)	3.0	(0.3, 15)	1.75	(0.3, 20)	2.0	(.3, 72)

^A^ p = 0.040 (2x2 chi-square, Fisher’s Exact Test)

^B^ p<0.001 (one-way ANOVA)

^C^ p = 0.052 (Independent Samples Median Test)

Family caregivers of people with primary brain cancer were mainly female (75.9%), with a mean age of 56.7 years (SD = 11.2), married (89.7%), retired (42.9%) and 86.2% were spouses/partners of the care recipients (Table 1). People with brain cancer were predominately male (72.4%) with a mean age of 60.6 years (SD = 10.75). They had a median time since diagnosis of 12.0 months (range 1–150) and a median length of receiving palliative care of 3 months (range 0.3–15). Compared to the group who had brain metastases and the group with all other cancers, the primary brain cancer group differed significantly in patient age being ten years younger (p<0.01), and more family caregivers were spouses (86% compared to 63–68%, p = 0.040) (Table 1). The two groups with brain metastases and all other cancers were closer in characteristics to each other than to the brain cancer group, therefore they were grouped together for the subsequent analyses.

### Comparative analysis of primary and secondary outcomes

For those who completed the study in the intervention group, Table 2 compares primary brain cancer (n = 13) scores with all other cancers (n = 201) scores (including brain metastases) at each time point, and the difference over time.

**Table 2:** Between group comparisons for outcome variables in the intervention group.

	Primary brain cancer	All other cancers	Difference between groups LSM [95% CI]	p-value
N	13	201		
FACQ-PC				
Caregiver Strain				
Baseline mean [SD]	3.35[0.86]	2.85[0.72]	0.49[0.082 to 0.906]	***0*.*019***
Follow up mean [SD]	3.31[0.94]	2.79[0.69]	0.52[0.118 to 0.916]	***0*.*011***
Mean change [SD]	-0.04[0.36]	-0.07[0.49]	-0.03[-0.300 to 0.242]	0.833
SF 12v2				
Mental Component Score				
Baseline mean [SD]	35.96[12.64]	43.66[10.18]	-7.71[-13.54 to -1.87]	***0*.*010***
Follow up mean [SD]	41.41[12.95]	44.82[10.47]	-3.41[-9.406 to 2.586]	0.264
Mean change [SD]	5.46[10.90]	1.25[9.80]	-4.21[-9.780 to 1.357]	0.137
Physical Component Score				
Baseline mean [SD]	58.22[6.71]	51.97[9.44]	6.25[2.042 to 10.46]	***0.006*** *
Follow up mean [SD]	54.18[6.60]	51.54[9.92]	2.64[-2.876 to 8.145]	0.347
Mean change [SD]	-4.04[8.41]	-0.49[6.56]	3.56[-0.215 to 7.327]	***0.009*** *
Caregiving workload				
ADL				
Baseline mean [SD]	2.35[1.06]	1.66[0.72]	0.68[0.039 to 1.327]	***0*.*039***
Follow up mean [SD]	2.21[0.92]	1.79[0.80]	0.41[-0.044 to 0.867]	0.077
Mean change [SD]	-0.14[0.65]	0.12[0.60]	0.26[-0.079 to 0.602]	0.132

Independent samples *t*-test

*non-parametric test (Mann-Whitney U Test)

In the primary outcome of caregiver strain, there was a significant difference between the two groups at baseline (p = 0.019) and follow up (p = 0.011), with the primary brain cancer group having higher levels of caregiver strain at both time periods. However, the change between baseline and follow up was very small for both groups and not significant (Table 2).

In the secondary outcome of mental wellbeing, there was a significant difference between the baseline scores of both groups (p = 0.010), with the primary brain cancer group having lower levels of mental wellbeing. There was an improvement in mental wellbeing over time in each group, but these improvements were not significant, however worth noting that the primary brain group had a greater improvement in mental wellbeing over time (Table 2).

For physical wellbeing, there was a significant difference between the baseline scores of both groups, with the primary brain cancer group having higher levels of physical wellness at baseline and follow up (possibly due to the younger age group). However, there was a reduction in physical wellbeing over time in each group, but these changes were not significant. The primary brain group had the greater reduction in physical wellness over time (p = 0.009) (Table 2).

With the outcome on caregiving workload (assisting with activities of daily living), there was a significant difference between the baseline scores for both groups (p = 0.039), with the primary brain cancer group having a higher level of ADL workload at baseline and follow up. However, it appears that the primary brain group had a slight reduction in ADL workload over time, while the other cancers group had a slight increase in ADL workload over time (Table 2).

### Family caregivers’ identified needs and solutions provided

**Fig 1:**
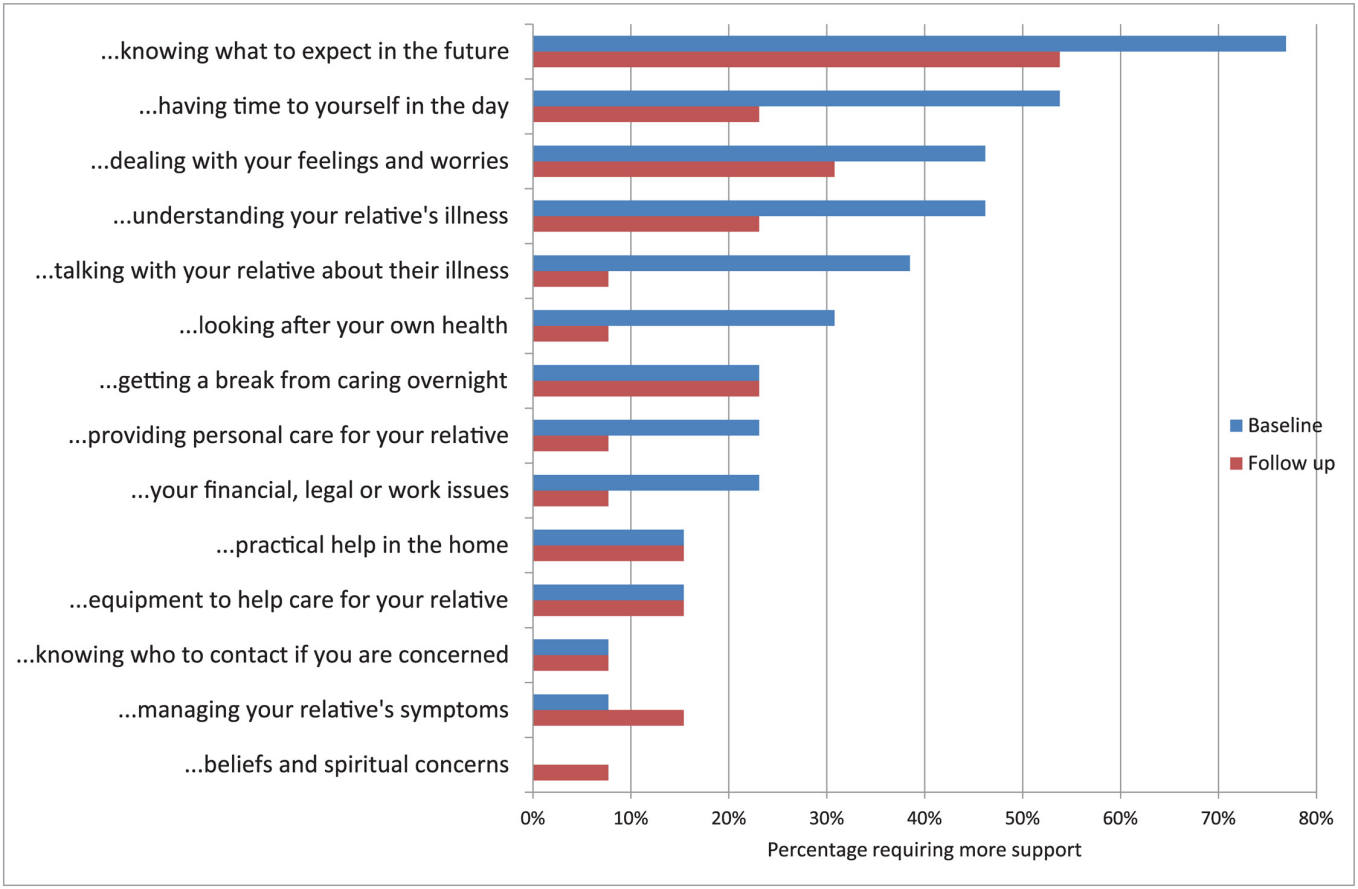
Percentage of the primary brain cancer group requiring more caregiving support at baseline and follow up, n = 13.

**Fig 2:**
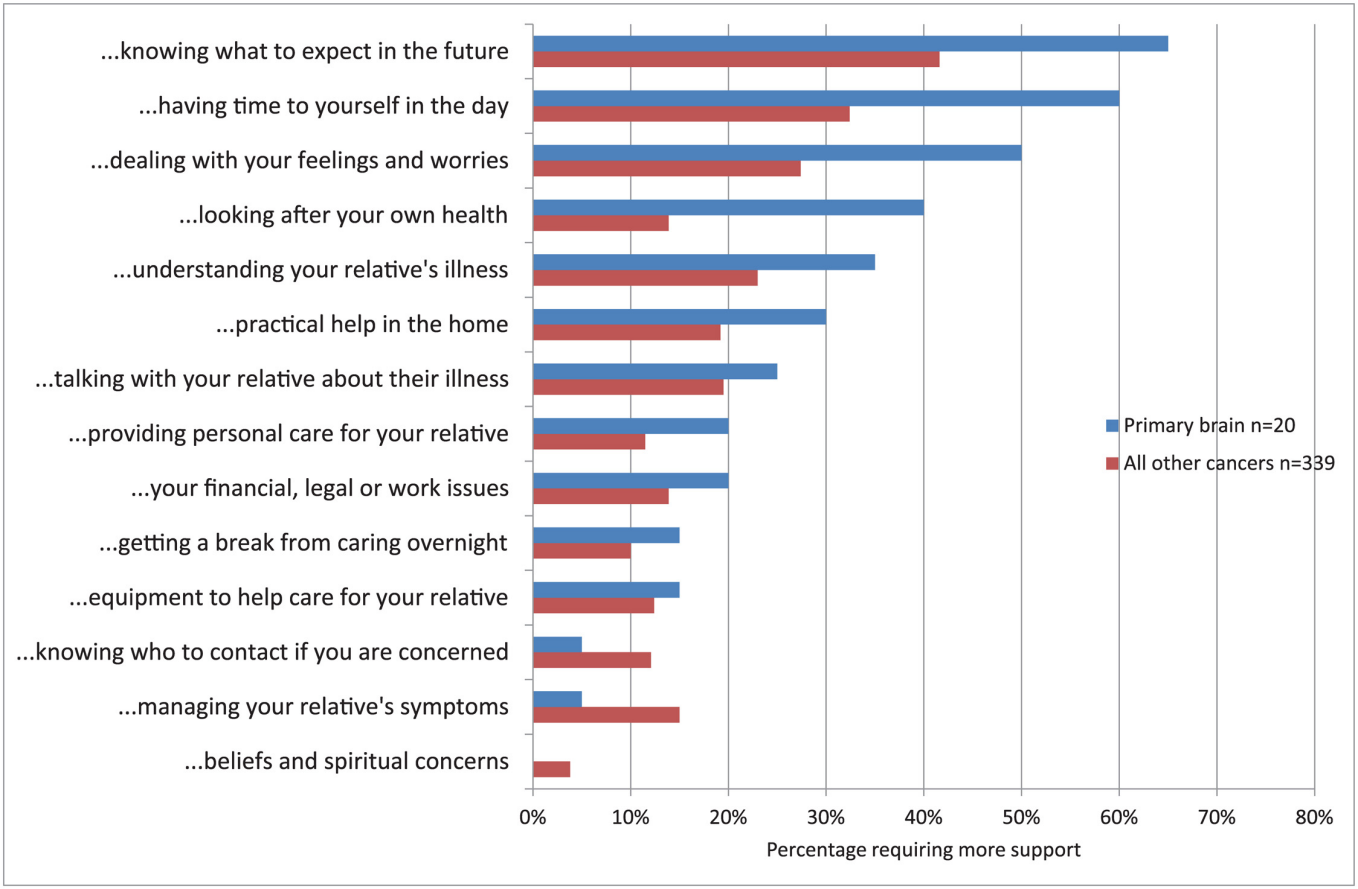
Percentage of the two cancer groups (brain cancer and all other cancers) requiring more caregiving support at baseline.

The top four support needs of primary brain cancer family caregivers reported at baseline were (Fig 1): knowing what to expect in the future (76%), having time for yourself in the day (53%), dealing with your feelings and worries (46%), and understanding your relative’s illness (46%). While most of those needs have decreased by the second follow up visit, the needs that stayed the same were “getting a break from caring”, “practical help in the home” and “equipment to help care”. One need was more pronounced at follow up and that is “managing your relative’s symptoms”, while one need surfaced at follow up and that is “beliefs and spiritual concerns”. Compared to the all other cancers group, the primary brain cancer group had more intense needs in all support needs except three, particularly in the top four priorities (Fig 2).

The solutions the nurses provided for “knowing what to expect in the future” consisted of ongoing discussions around the disease process, side-effects of treatment and end of life issues for home care versus hospice care, advance health directives and future care and the role of palliative care. Reassurance of further clarification, guidance, and increased support was provided from the nurses if deterioration occurred, however in one instance the caregiver was not wanting to know what is to come until things deteriorate, whilst another only wanting to discuss the positives and not the negatives. Regarding the second priority “having time for yourself in the day”, nurses referred to Social Workers, the Neurodegenerative Conditions Coordinated Care Program liaising with service providers to negotiate respite availability, including weekends and overnight, discussed strategies for creating more personal time for the caregiver despite the reluctance by some caregivers.

The solutions provided for the third priority “dealing with your feelings and worries” consisted of on-going education, discussions on prognosis and uncertainty, information on various avenues for counselling and encouragement or guidance regarding speaking to the oncologist. For the fourth priority “understanding your relative’s illness” nurses provided ongoing education, discussions about progress, arranging for a review by palliative care doctor when necessary, ensuring awareness of their inability to predict what is going to happen and providing guidance when a family caregiver and client are seeking another opinion about his/her diagnosis/treatment.

### Family caregivers’ experiences using the CSNAT assessment process

Four themes evolved from the family caregivers’ feedback interviews (Median = 17.5 minutes Range = 8–58 minutes): The extremely challenging caregiver experience with brain cancer; the systematic and practical approach of the CSNAT during rapid changes; connection with health professionals, feeling acknowledged and empowered; and timely advice and assurance of support during the caregiving journey.

### Theme 1: The extremely challenging caregiver experience with brain cancer

Caregivers provided feedback on the CSNAT assessment process and about their personal situation expressing their extremely challenging experience with brain cancer, undertaking demanding tasks whilst dealing with other family issues and responsibilities and often feeling “overwhelmed” (FC251): *"A tsunami of tragedies"*.*(FC251); “If people are made aware of the feelings carers are going through it may be beneficial in providing more support for others*.*”(FC017);* and, *“It makes you think a little—it made me realise the emotional side of caring and how a lot of those issues you tend to "soldier on"*. (FC115)

The personal demands of ‘soldering on’ throughout the caregiving journey encompassing psychosocial issues, was acknowledged by caregivers and articulated by this participant dealing with a move from a rural area to access treatment: *“Getting over the loss of your plans [is hard]*. *I'm very independent and at first I tried to do everything alone*, *and it felt at first I'd failed if I couldn't do everything*. *But now I've learnt from this and I've now "let people in” to help*.*”* (FC32)

The reluctance sometimes shown by caregivers to accept assistance from others was evident in the previous quote however that caregiver was subsequently prepared to allow people to help. A different barrier preventing a caregiver asking for help can be the difficulty of accessing resources whilst attending to caregiving demands, often struggling to cope alone until a crisis eventuates and they seek assistance: *“People are scared of accessing resources or unaware of who to call*. *A health crisis prompted me to ask for help*.*”(FC043)*

The need for more information was revealed as concerning for some caregivers, *“Any bit of information helps*.*”* (FC32), including basic physical care that family caregivers often feel unprepared for in their new role and responsibilities with the rapid onset of brain cancer:

*One thing I think could have been useful is some sort of training course or information on how to do basic things… we were doing it the wrong way*. *All sorts of basic "how to" things*, *that was what we really needed to know…*. *because we wouldn't have a clue*, *but when you see the [Silver Chain] carers do it you think "Oh we've been struggling to do that" & it's so easy when they showed us & it makes it better for the patient as well*.

*(FC325)*

Whilst caregivers described how the emotional aspects of their new role are often not recognised by themselves during the constant demands of attending to the physical needs of their relative, the CSNAT questions allowed them to reflect honestly about their situation:

*Emotionally—it makes you reflect where you are in process*. *A little confronting as you had to look at questions and be honest with yourself in where you are in process*. *Not the physical but the emotional aspect*. *You tend to get caught up in the physical aspect but the questions make you think about the emotional aspect*.

*(FC195)*

However with the focus of care being primarily on the person with brain cancer, unmet needs of the caregiver are often neglected by health professionals: *“No—they look more at the medical needs of the patient*.*”* (FC447)

### Theme 2: The systematic and practical approach of the CSNAT during rapid changes

The systematic format of the CSNAT and ease of completion was considered important by family caregivers of a person living with brain cancer as they often experience time constraints due to their challenges and competing demands. Family caregivers described using the CSNAT as *“Straight forward*. *Not too wordy*.*”* (FC382)*; “Very easy*, *I completed it by myself*.*”* (FC479) *and “Initially I completed it myself—I wanted to take my time & think about it*.*” (FC424)*

The structure of the assessment was appreciated by the family caregivers, providing an opportunity to discuss their needs as listed in the CSNAT: *“Going through the list—it gives a checklist*. *It makes you more aware of things—it's made me into a "list person"*. (FC424); “*Yes it underlined areas—you can go back to the paperwork later to refresh your memory and can work on [it] yourself*.*” (FC132)* and *“It made me think about a few things—going through the list” (FC343)*

The caregivers in this study considered it useful for themselves as well as for the nurses to consider the stage of the disease trajectory when completing the CSNAT. Caregivers commented on how their own needs can change quickly as their relative’s condition deteriorated: *“You can tell them [nurses] what you need and at first we didn't need much*, *but then the needs changed*. *It's good for you and good for us*.*”* (FC479), and *“Pleased to do them with a time gap between forms where my needs had changed*.*” (FC348)*. In the later stages of the disease different caregiver needs were expressed, requesting *“a few more questions about dealing with feelings and worries”*, and *“more information about relative's illness*. *You have different needs at different stages*.*”* (FC313)

The CSNAT assessment process was considered “*really thorough”* by Participant 424, however completing it earlier in the illness trajectory would have been more beneficial whilst dealing with the uncertainty of the prognosis and often rapid progression:

*Yes*. *By the time we filled in the forms we'd been through the worst of it*. *I think it would be better in the early stages when we had a lot of uncertainties… Is there some way carers could get some information early in the piece*?

(FC424)

### Theme 3: Connection with health professionals, feeling acknowledged and empowered

The assessment process acknowledged and validated the caregiving role as articulated by participants: “*Sometimes I think it [caregiving] goes unnoticed*. *Yes*, *it's totally acknowledged what you're doing…It's not something you want acknowledgement for*, *but different people have different ideas*.*” (FC343)*. The chance to acknowledge and discuss their personal situation with a health professional was considered important: *“You can talk about it to someone and you're not all on your own*.*”* (FC438)

The opportunity to connect or bond with the nurses whilst completing the CSNAT was valued by caregivers during what is often a very isolating journey:

*“Taking time to realise what you're doing [caregiving]—by doing this research even Silver Chain can get to know what's inside the carer's head*. *Taking time to go through the forms you have the opportunity to bond [with the nurses]*.*”*

(FC424)

*Yes*, *doing the form and having the nurse coming to the house—it made you make the time to deal with things*.

*(FC132)*

*I imagine it will benefit others in the future*. *To see how carers feel—working on what they need*. *You as carer can be a medium between the nurse and the patient when the patient is too sick to voice their needs*.

*(FC348)*

As a caregiver-led assessment process, the CSNAT allowed caregivers to reflect on what they needed “*Yes*. *Questions were all about the carer rather than the patient” (FC313)*, or could do themselves following a discussion facilitated by the nurse: *“I’m going to see counsellor as result”* (FC134). The process of completing the CSNAT provided an opportunity for the health professional to identify the range of support available to meet a caregiver’s needs:

*“Yes*, *on the last one [CSNAT]—I felt I'd had all the support I needed*, *but the nurse explained I could get help with the housework*. *So I was then the wiser for what help was available*.*”*

(FC416)

Associated with the CSNAT process, the discussion between the nurse and caregiver resulted in empowering caregivers in their new role to find solutions themselves: *“It got you talking*. *Yes—it highlighted the areas you needed to get help or work on yourself”* (FC132); *“Yeah*, *it helped a bit in getting support*. *It pointed me in the right direction*.*”* (FC348), and *“Personally*, *being able to discuss the issues was very beneficial*. *To highlight who I need to go to*. *I know who to contact in the future—forward planning*!*” (FC132)*

A personalised approach to address the practical and spiritual needs of Caregiver 461 demonstrates how positive outcomes resulted from completing the CSNAT with a health professional who connected to the individual’s identified needs and tailored the appropriate support. Following the first CSNAT this caregiver was provided with practical support from home cleaning, emotional support with counselling and spiritual support though meditation, a complementary therapy provided by a local Community Hospice:

*When I completed the first [CSNAT] form–[SC Home Help] is coming to clean now*. *Footprints [Day Centre] phoned and I started a session [for meditation]*. *A counsellor came and was helpful and now I don't need a [religious] minister to come*.

*(FC461)*

### Theme 4: Individualised and assurance of timely support during the caregiving journey

Reassurance resulted from the assessment process when caregivers received the expertise and support provided by nurses and is evident in these responses: *“Yes*, *every time they [nurses] visit they ask how I'm going*.*”* (FC504) Various support and reassurance was gained for this family caregiver: *“Oh yes*. *The doctor & people are reassuring us we are doing quite well to get him to this stage*. *I really do feel I have the support of my family and the nurses*.*” (FC299)*

Adjustments to work commitments can cause distress to family caregivers of people with brain cancer with the uncertainty of the disease trajectory and high levels of care required. Following the CSNAT process for this caregiver with a remote mining job requiring periods away from home, an Aged Care Assessment was arranged and high level home care approved, alleviating the stressful situation:

*I’m just very grateful for the offer that we’re getting…If we can keep my wife in the house initially that will be great and I think that's what we're trying to do*. *It was getting to the point where I was going to have to decide between caring for my wife or work*, *but if I don't work it wouldn't be very long before I couldn't support my wife*.

*(FC343)*

In some instances just the CSNAT assessment process itself, ‘flagging’ their issues to the nurse during a discussion was considered by caregivers as providing them the necessary support *“Yes*. *I knew some things I couldn't get help with—but just talking*, *flagging or raising issues was useful*.*” (FC134)*

*It made me more aware*. *You don't know about these things until they directly affect you*. *You go wandering through your life in total ignorance until it all goes wrong*, *and then when it goes wrong is only when you find out about things*. *But it definitely helped*.

*(FC297)*

A family caregiver may experience a sense of being overwhelmed during their numerous roles and responsibilities whilst caring for a person with brain cancer. Timely advice and support during the CSNAT process was appreciated by Family Caregiver 348 and eased their need for further time consuming communications: *“It helped—it answered questions I had and saved me from making unnecessary phone calls to [service]–the nurse sat and discussed things then and there*.*”(FC348)*

*Yes*, *they provided a counsellor—it was very helpful to have a contact number*. *The nurse provided a booklet on what to expect in the future—very helpful*. *I liked that you have to point 3 most important needs- and [service] offered help*.

*(FC404)*

The relief experienced through receiving practical support to alleviate the overwhelming pressure of the demands of caregiving was pertinently expressed by Family Caregiver 416:

*It takes away the overwhelming feeling*. *Knowing the help (cleaning)*, *for someone who has everything immaculate to saying*, *‘that doesn't matter’—subconsciously you have a ‘little birdie' talking at you—you need to do this’*. *It's overwhelming the thought of doing it*, *so suddenly from being overwhelmed to thinking ‘I'm going to have some help with this’*. *It was a relief of pressures ‘like a little pressure valve released'*.

*(FC416)*

## Discussion and Conclusion

This is an exploratory study drawing data from a rigorous stepped wedge cluster trial in community palliative care. Both the quantitative and qualitative analyses are of complementary value. In the quantitative section of the study, the authors identified and reported the higher burden placed upon caregivers of brain cancer patients compared to other cancer groups. The qualitative aspect of the study provided important insight as to how this burden is manifested through interviews with family caregivers and their personal quotes.

Comparing the baseline characteristics between the caregiver groups, it was evident that caregivers of the brain cancer group significantly experienced higher strain and workload and lower mental wellbeing than the group of all cancers. Although, the primary brain cancer group had higher physical wellbeing at baseline, possibly due to the younger age group of both patients (p<0.001) and caregivers, they had the greater reduction in physical wellness over time. All other differences over time were not significant and the sample size of the brain cancer group was too small to detect such differences.

However the higher self-reported burden of this group may indicate that brain cancer caregivers (higher proportion of spouses, p = 0.04) spend more time on daily caring activities in the longer term compared to other caregivers. Brain cancer patients in this study have being longer with the palliative care service compared to other patients (median 3 months compared to 1.75–2 months, p = 0.052). Moreover behavioural problems are known to be strong predictors of caregiver burden [6,8,20] and caregivers may be less likely to obtain appropriate and timely support [13,15,22]. In their qualitative feedback, caregivers commented on the need to go through the CSNAT earlier in their disease journey to access information, training and other practical and timely supports before the end of life stage.

Information needs for brain cancer family caregivers are well documented, [7,13,15,19,22] and the CSNAT provided an assessment process for these needs to be addressed. Research shows family caregivers of people with brain cancer want access to communication with health professionals to discuss their concerns as they navigate the challenging disease trajectory [13,15,19,22,31]. Furthermore, Collins et al. [6] recommended an on-going ‘therapeutic relationship’ be developed with the family caregiver, facilitating an individualised needs assessment, planning for future needs to avoid crisis-led support (echoed in other palliative care research) [32]. By completing the CSNAT, caregivers were able to discuss any issues and concerns face-to-face with their regular hospice nurse and identify their personal needs. Thus in contrast to other studies, this study provided opportunities for identified issues of concern to be addressed from a practical perspective.

Reviewing caregivers’ needs 2–3 weeks apart provided evidence about the benefit of systematically using the CSNAT approach. As shown in other CSNAT studies [14,25,26], knowing what to expect as the illness progresses continued to be the top priority at baseline and follow-up, indicating the need to provide information frequently during the disease trajectory, and on-going support for caregivers’ concerns about the future. While it is clear that caregivers frequently request the need for more information, the precise nature of the information and the way it is optimally presented deserves further study.

Neuro-behavioural changes are extraordinarily challenging for people with brain cancer and their family caregivers [6,8,15,20] compared to most other life-limiting diseases, especially as rapid deterioration often occurs and symptoms include neurocognitive impairment and language difficulties. Furthermore, there is an additional psychological burden of care upon their family caregivers, as the patients may experience unexpected events with a sudden alteration in conscious state or clinical behaviour, at times with catastrophic consequences e.g. brain haemorrhage, seizures etc. [6–8,15]. A recent study suggests health professionals need to reconsider the patient-centered model of care to integrate a collaborative approach of care coordination into their routine practice of a partnership for brain cancer patients and their caregivers [6]. As their partners’ personality changes or behavioural changes develop or cognitive ability to communicate diminishes, stress relating to the changed ‘essence’ of relationships can be experienced by brain cancer caregivers [6,8,22]. Moreover, the wider family can be affected by the caregiver’s overwhelming challenges, whilst balancing competing demands and roles [18,20].

From a practical perspective, clinicians may easily perceive the distress which caregivers of their patients may be experiencing but yet find it challenging to clearly identify the precise physical and psychological factors which are contributing to the distressed state. On the other hand, caregivers themselves may find it difficult to articulate their concerns in a manner which may result in useful intervention [26]. However the CSNAT’s succinct and systematic approach provided participants with an opportunity to share their challenging experiences, gaining acknowledgment of their role as a caregiver, insight into their psychosocial needs and awareness of available support. Using such a caregiver-led assessment process for identifying and addressing family caregivers’ support needs, the CSNAT approach gave them a sense of validation, reassurance and empowerment, as reflected by their quotes.

In keeping with results from caregivers in other cancer fields using the CSNAT [25], support needs primarily relating to ‘direct’ support are reported as priorities by brain cancer caregivers, such as “dealing with your feelings and worries” and “having time for yourself in the day”. Supportive interventions were considered beneficial earlier in the caregiving trajectory by brain cancer caregivers in this study, as well as previous family caregiving research in the cancer field [26] and the MND field [33]. Collins et al. [6] suggest an early integration into palliative care for brain cancer caregivers to provide a coordinated support.

## Limitations

The small sample size of the brain cancer group and the difference in sizes between the two groups pose limitations that preclude detailed analyses. However, an important goal of this pilot study was to ascertain the feasibility of a larger trial. Both the quantitative and qualitative findings confirm the need for a larger study, which would allow conclusions with a greater clinical impact to be drawn. In their meta analyses on caregiver burden interventions, Northouse et al [34] concluded that even though effects are generally small to moderate in magnitude, these interventions can significantly reduce caregivers’ burden and lead to positive outcomes “as these interventions produce more prepared, less distressed caregivers which in turn is likely to result in more positive benefits for patients”.

## Conclusion

The results of this initial study indicate that the CSNAT provides a concise and validated approach to identifying the concerns of caregivers of brain cancer patients in a community-based palliative care setting. Furthermore, despite the relatively small sample of patients, the current study has demonstrated that this supportive intervention may be delivered to family caregivers in a feasible and beneficial way. A larger scale, longitudinal study is therefore being planned for recruitment of patients following the initial diagnosis of primary and secondary malignant brain tumours. This approach would potentially allow a more detailed appreciation and characterisation of the evolving caregiver needs at different stages of the disease. The data would provide an evidence base for the future planning and coordination of services, ensuring that services are tailored specifically to the individualised and ‘unique’ support needs of family caregivers of people with brain cancer.
