# Correction: Exploring the Support Needs of Family Caregivers of Patients with Brain Cancer Using the CSNAT: A Comparative Study with Other Cancer Groups

**DOI:** 10.1371/journal.pone.0148074

**Published:** 2016-01-22

**Authors:** Samar M. Aoun, Kathleen Deas, Denise Howting, Gabriel Lee

There is an error in the third sentence of the Abstract. The name of the tool is incorrect. The correct sentence is: The Carer Support Needs Assessment Tool (CSNAT) is a validated instrument designed to systematically identify and address caregiver needs.

## References

[1] Aoun SM, Deas K, Howting D, Lee G (2015) Exploring the Support Needs of Family Caregivers of Patients with Brain Cancer Using the CSNAT: A Comparative Study with Other Cancer Groups. PLoS ONE 10(12): e0145106 doi:10.1371/journal.pone.0145106 26679505 10.1371/journal.pone.0145106 PMC4682982

